# Waste Plastic-Supported Pd Single-Atom Catalyst for Hydrogenation

**DOI:** 10.3390/ma17133058

**Published:** 2024-06-21

**Authors:** Ziyue Wang, Ying Zhang, Hao Zhang, Qingdi Sun, Xiaohui He, Hongbing Ji

**Affiliations:** 1 Key Laboratory of Bioinorganic and Synthetic Chemistry of Ministry of Education, Fine Chemical Industry Research Institute, School of Chemistry, Institute of Green Chemistry and Molecular Engineering, Sun Yat-sen University, Guangzhou 510275, China; wangzy258@mail2.sysu.edu.cn (Z.W.); zhangy656@mail2.sysu.edu.cn (Y.Z.); zhangh577@mail2.sysu.edu.cn (H.Z.); sunqd@mail2.sysu.edu.cn (Q.S.); 2 Guangdong Technology Research Center for Synthesis and Separation of Thermosensitive Chemicals, Guangzhou 510275, China; 3 State Key Laboratory Breeding Base of Green-Chemical Synthesis Technology, Institute of Green Petroleum Processing and Light Hydrocarbon Conversion, College of Chemical Engineering, Zhejiang University of Technology, Hangzhou 310014, China

**Keywords:** waste plastics, single-atom catalyst, hydrogenation, palladium, rapid thermal processing reactor

## Abstract

As worldwide plastic pollution continues to rise, innovative ideas for effective reuse and recycling of waste plastic are needed. Single-atom catalysts (SACs), which are known for their high activity and selectivity, present unique advantages in facilitating plastic degradation and conversion. Waste plastic can be used as a support or raw material to create SACs, which reduces waste generation while simultaneously utilizing waste as a resource. This work successfully utilized waste plastic polyurethane (PU) as a support, through a unique Rapid Thermal Processing Reactor (RTPR) to synthesize an efficient Pd_1_/PU SACs. At 25 °C and 0.5 MPa H_2_, Pd_1_/PU displayed outstanding activity and selectivity in the hydrogenation of styrene, as well as remarkable stability. Pd_1_/PU performed well in hydrogenating a variety of common substrates. These findings highlight the great potential of SACs in plastic waste reuse and recycling, offering intriguing solutions to the global plastic pollution problem.

## 1. Introduction

Catalysts with high efficiency, particularly single-atom catalysts (SACs), have become critical in modern energy and chemical industries, showcasing significant catalytic performance in various key industrial reactions such as selective hydrogenation [1,2,3,4,5,6], selective oxidation [7,8,9], dehydrogenation [10], hydrogen production [11,12], various coupling reactions [13,14,15], and classic electrochemical processes [16,17,18,19]. SACs, with high atomic utilization (~100%) and well-defined electronic geometric structures [20,21,22,23,24,25], are suitable for investigating the catalytic mechanisms and bridging the gap between homogeneous and heterogeneous catalysis [26]. In catalytic hydrogenation, catalysts with metals such as Pd, Pt, Au, and Ru, alongside metal oxide materials like TiO_2_, Cu_2_O, and CeO_2_ [27], display a broad range of applicability and notable advantages. The exceptional properties and catalytic performances of these materials are garnering increasing attention. Polymer-loaded metal or metal oxide nanoparticles exhibit significant potential and versatility in catalytic hydrogenation [28]. These catalytic materials not only possess unique catalytic properties, but also offer enhanced stability and recyclability, thereby broadening the application scope of catalytic hydrogenation reactions. Apart from their efficacy in the manufacture of bulk chemicals, fine chemicals, and pharmaceuticals, these catalysts also present novel applications in the recycling and reuse of plastic waste [29].

Plastic is indispensable in modern life because of its affordability and exceptional longevity, making it the preferred option for various uses ranging from domestic goods to industrial equipment [30]. The increase in plastic manufacturing has caused a surge in plastic pollution, posing a significant issue for contemporary civilization [31,32,33]. Polyurethane (PU) is a polymer with many urethane linkages (–NH–COO–) that is highly valued in various industries for its outstanding chemical stability and gradual degradation [34,35,36,37]. However, these traits impede the organic breakdown of PU in the environment, leading to significant ecological issues [38]. While traditional infinite insulating materials (such as silica or alumina aerogels), excel as carriers [39,40], this study aimed to tap into the significant potential of SACs in plastic pollution control by utilizing plastic waste as a carrier.

We employed a Rapid Thermal Processing Reactor (RTPR) to address the difficulties associated with PU recycling. In conventional thermal processes, the low melting point of the PU support often leads to melting, which restricts the efficacy and stability of the catalyst. To overcome this challenge, a RTPR was employed, which rapidly reaches the target temperature within a very short time. This rapid calcination process ensures that the active metal Pd sites are securely anchored while maintaining the structural integrity of the PU support [41]. In addition, the porous structure of waste plastic PU as supports with stable urethane linkages provides a unique entry point for the local coordination chemistry of single atoms at the center of SACs. The RTPR is expected to create new opportunities for recycling and reusing waste plastics by merging the high efficiency and atomic-level dispersion of SACs with the stable structure of waste plastic PU. This method promotes resource recycling, diminishes environmental degradation, and could create fresh revenue prospects for SACs. The future of using RTPR in PU recycling looks very promising due to advancements in research and technology.

As described previously, Pd_1_/PU catalysts were successfully synthesized with the support of PU, utilizing RTPR. Encouragingly, the Pd_1_/PU prepared using RTPR showed no significant changes before and after calcination. The Pd_1_/PU exhibited excellent activity (97%) and selectivity (99%) under the hydrogenation of styrene at 25 °C and 0.5 MPa H_2_ for 15 min, and also demonstrated outstanding stability, with minimal decreases in activity and selectivity after six reaction cycles. Additionally, the Pd_1_/PU was effective in hydrogenating a series of typical substrates. The H_2_-D_2_ exchange experiments further verified the higher hydrogenation activity of Pd_1_/PU SACs compared to Pd_n_/PU nanoparticles. This is due to the distinctive single-atom structure, which aids in H_2_ dissociation and significantly enhances catalytic activity. This work provides a new perspective and strategy for recycling and reusing waste plastics, highlighting the significant potential of SACs in the field of environmental protection and sustainable development.

## 2. Materials and Methods

### 2.1. Materials

Polyurethane (PU; Elastollan^®^ A C 80 A HPM (LP 9307)) was purchased from BASF. (Shanghai, China) Tetraamminepalladium(II) nitrate [Pd(NH_3_)_4_(NO_3_)_2_] (CAS: 13601-08-6) and allylbenzene (CAS: 300-57-2) were purchased from Shanghai Macklin Biochemical Co., Ltd. (Shanghai, China) Styrene (CAS: 100-42-5), methanol (HPLC grade; CAS: 67-56-1), ethyl alcohol (CAS: 64-17-5), isopropanol (CAS: 67-63-0), 4-bromostyrene (CAS: 2039-82-9), and 4-ethenyl-benzenamine (CAS: 14235-81-5) were purchased from Energy Chemical. (Shanghai, China) Tridecane (CAS: 629-50-5), 1-hexene (CAS: 592-41-6), cyclohexene (CAS: 110-83-8), 3-ethenyl-benzenamine (CAS: 15411-43-5), and 1-chloro-4-ethenylbenzene (CAS: 1073-67-2) were purchased from Aladdin Co., Ltd. (Shanghai, China) H_2_/N_2_ (5%) and D_2_ (99.999%) were purchased from Guangzhou Guangqi Gas Co., Ltd. (Guangzhou, China)

### 2.2. Catalyst Preparation

To synthesize Pd_1_/PU, PU (0.5 g) was initially dispersed ultrasonically in 150 mL of deionized water for 15 min. Pd(NH_3_)_4_(NO_3_)_2_ was then dissolved in 100 mL of deionized water and subjected to ultrasonic treatment for 15 min. Following that, a precursor solution was added to the PU suspension one drop at a time, while vigorously swirling at 25 °C. After 4 h of aging, the precipitate was filtered through a Büchner funnel, washed three times with 1 mL of methanol, and dried at 60 °C for 12 h. Lastly, the pre-prepared sample was placed in a quartz tube under a continuous nitrogen flow rate of 30 mL/min, and then placed in a tube furnace preheated to the required temperature of 300 °C. The sample produced was maintained at 300 °C for one minute. The quartz tube was then quickly removed and swiftly cooled to room temperature, resulting in the formation of Pd_1_/PU.

To synthesize Pd_n_/PU, Pd(NH_3_)_4_(NO_3_)_2_ was dissolved in 2.5 mL of deionized water and ultrasonicated for 15 min to reach the required volume for impregnating 0.5 g of PU. The precursor solution was then mixed with PU. After 4 h of aging, the sample was dried at 60 °C until fully dehydrated. Following the same calcination process as Pd_1_/PU, Pd_n_/PU was obtained by the incipient wetness impregnation method.

### 2.3. Catalyst Characterization

The metal contents of each catalyst were analyzed using inductively coupled plasma mass spectrometry (ICP-MS, Agilent 7700, Santa Clara, CA, USA). A Cu Kα (λ = 0.15432 nm) ray was used to capture the X-ray diffraction patterns using a LIFM-X-ray powder diffractometer (Smart Lab, Tokyo, Japan). Using MDI Jade 6 software, the data were processed and compared to PDF cards. Fourier transform infrared spectroscopy (FI-IR) spectra were acquired using the Nicolet iS20 spectrometer. (Waltham, MA, USA) The valence state of Pd_1_/PU SACs was examined using X-ray photoelectron spectroscopy (Thermo Fisher Nexsa-ALPHA, Waltham, MA, USA), and the results were processed with the XPSPEAK software (version 4.1). Transmission electron microscopy (TEM) and scanning transmission electron microscopy (STEM) images were obtained using a 300 kV acceleration voltage on an FEI Tecnai G2 F30 transmission electron microscope. (Tokyo, Japan) Aberration-corrected high-angle annular darkfield scanning transmission electron microscopy (AC HAADFSTEM) and energy dispersion spectrum (EDS) images were captured using a JEM-ARM200F transmission electron microscope operating at an acceleration voltage of 200 kV, equipped with double spherical aberration correctors. (Tokyo, Japan) The H_2_-D_2_ exchange experiments took place in a 7 mm inner diameter continuously flowing fixed-bed quartz reactor at room temperature and standard atmospheric pressure. Approximately 100 mg of catalyst was placed in the middle of the reactor with quartz cotton plugs. The carrier gas was a mixture of 5% H_2_/N_2,_ flowing at 20 mL/min, while D_2_ was employed as the pulse gas flowing at 10 mL/min. The HD (*m*/*z* = 3) signal was acquired in real time using the mass spectrometry (Hiden Analytical HPR-20 QIC benchtop gas analysis system, Manchester, UK).

### 2.4. Catalytic Performance Test

The styrene hydrogenation reaction proceeded in the following manner: 10 mg of catalyst, 0.18 mmol of substrate, 0.13 mmol of tridecane, and 2 mL of methanol were combined in a 10 mL stainless steel reactor. The reactor underwent five cycles of hydrogen (H_2_) purging. The reaction occurred at 25 °C with a reaction time of 15 min under 0.5 MPa H_2_. Once the reaction was complete, the liquid underwent filtration and was analyzed using GC (Shimadzu GC-2010 Plus, Kyoto, Japan). Its composition was verified using GC-MS (Shimadzu GCMS-QP2010 Ultra, Kyoto, Japan).

The stability of Pd_1_/PU SACs was tested in the following manner: 10 mg of catalyst, 0.18 mmol of substrate, 0.13 mmol of tridecane, and 2 mL of methanol were combined in a 10 mL stainless steel reactor. The reactor underwent five cycles of hydrogen (H_2_) purging. The reaction took place at 25 °C for one minute under 0.5 MPa H_2_. Once the reaction was complete, the liquid underwent filtration and was analyzed using GC (Shimadzu GC-2010 Plus). Its composition was verified using GC-MS (Shimadzu GCMS-QP2010 Ultra). The catalysts were retrieved through filtering and rinsed five times using deionized water and ethanol. The regeneration of the catalyst was achieved via filtration and drying at 60 °C for 12 h.

The conversion and selectivity of the reaction are, respectively, calculated as follows:

$$\begin{array}{c} \mathrm{Conversion}=\frac{c_{0}-c_{1}}{c_{0}}\times 100\%\\ \mathrm{Selectivity}=\frac{c_{2}}{c_{0}-c_{1}}\times 100\% \end{array}$$

*C*_0_ is the concentration of the reactants at the start of the reaction, *C*_1_ is the concentration of the reactants following the reaction, and *C*_2_ is the concentration of ethylbenzene following the reaction.

## 3. Results and Discussion

### 3.1. Synthesis and Characterization of Catalysts

**Figure 1:**
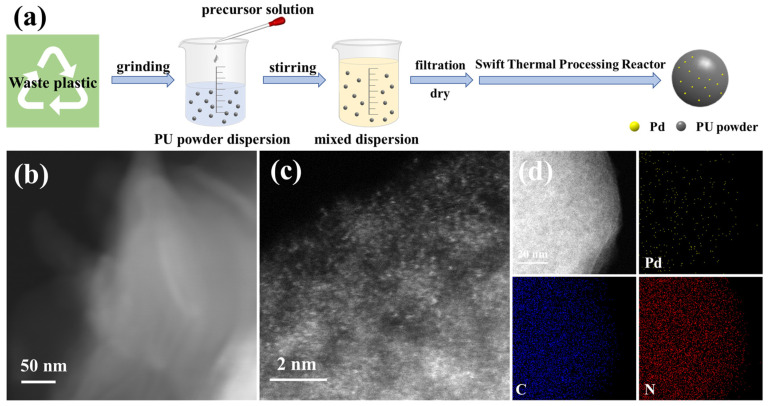
(**a**) Schematic diagram of the preparation process for Pd_1_/PU; (**b**) TEM image; (**c**) AC HAADF-STEM image; (**d**) EDS elemental mapping of Pd_1_/PU.

The goal was to recycle waste plastic by grinding it into powder for the purpose of creating catalytic supports. Using commercially available PU as the support, the process of synthesizing Pd_1_/PU SACs via the simple impregnation method and RTPR was carried out, as illustrated in Figure 1a and Figure S1. Usually, 1.4 mg of Pd(NH_3_)_4_(NO_3_)_2_ was mixed with 0.5 g of PU, followed by simple impregnation. The pre-prepared samples were rapidly heated to 300 °C within 3 min using RTPR, followed by intermittent calcination for 1 min, which facilitated the formation of Pd_1_/PU. Then Pd_n_/PU NPs were synthesized using the same heat-treatment methodology, along with the incipient wetness impregnation method.

**Figure 2:**
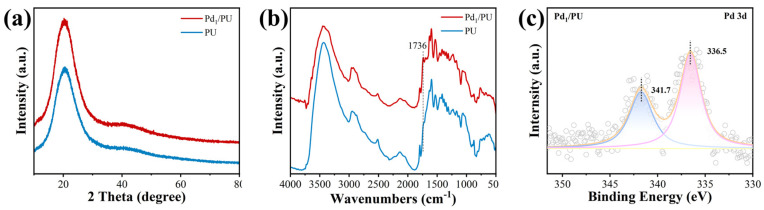
(**a**) XRD spectra and (**b**) FT-IR spectra of Pd_1_/PU and PU. (**c**) XPS spectra of Pd 3d in Pd_1_/PU.

The Pd content in Pd_1_/PU was determined to be 0.01 wt% using ICP-MS. There were no Pd nanoparticles or clusters detected in the TEM image of Pd_1_/PU (Figure 1b), which illustrates that the Pd species possibly existed as single atom. The Pd_1_/PU SACs were characterized using AC HAADF-STEM, revealing Pd single atoms as bright spots, as shown in Figure 1c. This highlighted the distinct benefit of RTPR in inhibiting the clustering of single metal atoms. In addition, the EDS elemental mapping verified that Pd species were uniformly distributed on the support as isolated atoms. (Figure 1d). The XRD analysis of Pd_1_/PU did not detect any standard peaks associated with the presence of Pd in a monatomic form, which is consistent with the HAADF-STEM data (Figure 2a). The presence of urethane linkages was confirmed by the peak at 1726 cm^−1^ in the FI-IR spectra (Figure 2b) and there were no other functional groups in the studied polyurethanes that could significantly affect the catalyst performance [42,43,44,45,46]. Furthermore, there was no substantial alteration in the structural attributes of the catalysts prior to and subsequent to calcination. This conclusively demonstrates the durability and efficacy of RTPR throughout the preparatory phase. In addition, peaks at 341.7 and 336.5 eV in the XPS spectra (Figure 2c) of Pd_1_/PU were identified as Pd^2+^ 3d_3/2_ and Pd^2+^ 3d_5/2_, respectively [47,48,49,50]. The results indicate that the Pd_1_/PU SACs were successfully synthesized using RTPR. An additional examination of the RTPR in the regulated synthesis of low-melting-point supports presents an unprecedented method for modifying the coordination chemistry at the site of a single atom in the center of SACs.

### 3.2. Catalytic Performance Test

**Figure 3:**
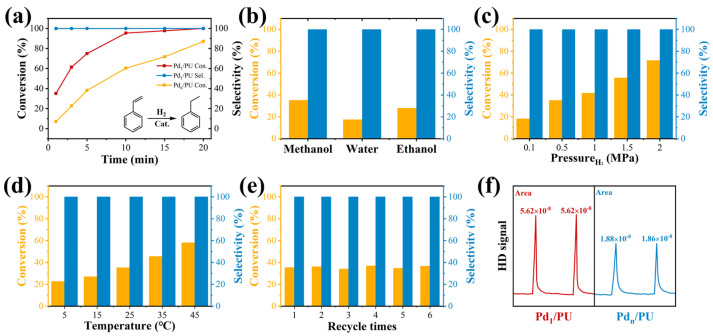
Reaction conditions: 2 mL of methanol, 0.18 mmol of substrates, 0.13 mmol of tridecane, 25 °C and 0.5 MPa H_2_; (**a**) time dependence of styrene conversion over Pd_1_/PU and Pd_n_/PU; (**b**) styrene conversion and selectivity in different solvents; styrene conversion and selectivity at (**c**) different pressures and (**d**) temperatures, respectively; (**e**) stability test on Pd_1_/PU in 6 cycles; (**f**) H_2_-D_2_ exchange experiments over Pd_1_/PU and Pd_n_/PU.

To evaluate the catalytic performance of Pd_1_/PU, the hydrogenation of styrene was assessed as a probe reaction. The work was conducted under 0.5 MPa H_2_ at 25 °C to evaluate the effect of reaction time on catalytic activity while balancing high conversion and reaction costs (Figure 3a). The results showed that Pd_1_/PU achieved a styrene conversion rate of 97.3% within 15 min, demonstrating higher hydrogenation activity of the SACs compared to the 71.7% conversion rate of Pd_n_/PU. The effect of the solvent on the reaction is illustrated in Figure 3b. Optimal performance was observed in the use of methanol. As a polar solvent, methanol can enhance the interaction between the substrate and the catalytic surface, facilitating the adsorption and activation of substrate molecules [51,52]. Methanol has a higher hydrogen bond-donating (HBD) capacity (α) than ethanol and therefore has more reactive activity [53,54]. Water, being a proton solvent, may compete with the catalyst and substrates for adsorption, potentially affecting the efficiency of catalytic reactions, requiring better substrate adsorption on the catalyst surface [55]. The effects of different pressures and temperatures on the activity of Pd_1_/PU were also studied, with the corresponding data presented in Figure 3c,d. The results indicate an increase in the reaction rate with higher temperatures and pressures. This is attributed to the fact that higher temperature provided more thermal energy, which enabled more molecules to overcome the activation energy barrier and increase the frequency of molecular collisions. Higher pressure also increased the concentration of reacting substances in the gas phase, which led to more frequent molecular collisions. The Pd_1_/PU SACs also exhibited excellent catalytic stability. As shown in Figure 3e, the catalytic performance remained consistent after six reaction cycles, with a conversion rate ranging from approximately 35.4% to 36.6%. The TEM image of Pd_1_/PU after six cycles (Figure S2a) did not show any Pd nanoparticles or clusters, indicating that Pd species likely existed as single atoms. According to AC HAADF-STEM, there were no Pd nanoparticles in the spent catalysts after six cycles and Pd remained in the form of single atoms, identified as individual bright spots (Figure S2).

Additionally, the hydrogen dissociation abilities of Pd_1_/PU and Pd_n_/PU were investigated in H_2_-D_2_ exchange experiments. As shown in Figure 3f, the peak area of Pd_1_/PU is approximately three times that of Pd_n_/PU, indicating that Pd_1_/PU SACs exhibit a stronger H_2_ dissociation ability compared to Pd_n_/PU. Consistent with the trend of hydrogenation activity (Figure 3a), the hydrogenation rate of Pd_1_/PU was greater than Pd_n_/PU under the same conditions. The single-atom form of Pd was more active than Pd in nanoparticle form, which is attributed to the fact that active sites were maximally exposed through uniform dispersion, resulting in higher hydrogenation activity of Pd_1_/PU SACs [28,56]. A variety of substrates were tested to determine the general applicability of Pd_1_/PU in hydrogenating C=C bonds (Table 1). All results demonstrated that Pd_1_/PU SACs showed excellent catalytic hydrogenation activity in different substrates at 25 °C and 0.5 MPa H_2_, which highlighted the exceptional scalability of Pd_1_/PU in the catalytic hydrogenation of C=C.

## 4. Conclusions

In summary, Pd_1_/PU SACs were successfully prepared using waste plastic as the support, through the unique RTPR method, resulting in highly efficient, sustainable, and cost-effective multiphase SACs. The hydrogenation of styrene obtained a conversion rate of up to 97% within 15 min at 25 °C and 0.5 MPa H_2_, with sustained high activity throughout multiple cycles. Pd_1_/PU SACs also showed an excellent hydrogenation range in compounds with C=C bonds. By combining the efficiency and atomic dispersion of SACs with the stable structure of waste plastic PU, this work aims to pave the way for new avenues in the recycling and reuse of waste plastics. This technology not only helps address plastic pollution and achieve resource recycling, but also has the potential to expand the application of SACs into new market sectors.

## Figures and Tables

**Table 1:** Catalytic hydrogenation of various C=C bonds with the Pd_1_/PU Catalyst ^a^.

Substrates	Products	Time (min)	Conversion (%)	Selectivity (%)
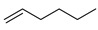	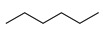	60	95.8	100
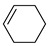	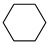	50	93.7	>99
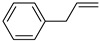	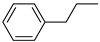	15	98.1	>99
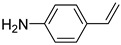	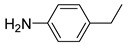	30	95.1	>99
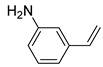	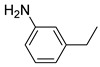	15	97.4	>99
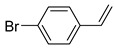	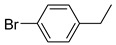	20	97.3	>99
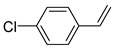	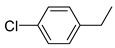	20	>99	88.4

^a^ Reaction conditions: 10 mg of catalyst, 0.10 mmol of the substrate, 0.5 MPa H_2_, and 2 mL of methanol at 25 °C.

## Data Availability

The raw data supporting the conclusions of this article will be made available by the authors on request.

## Supplementary Materials

The following supporting information can be downloaded at: https://www.mdpi.com/article/10.3390/ma17133058/s1, Figure S1: Schematic diagram of the Rapid Thermal Processing Reactor (RTPR); Figure S2: (a) TEM image and (b) AC HAADF-STEM image of Pd1/PU after six cycles.
