# Supplementary material for: Waste Plastic-Supported Pd Single-Atom Catalyst for Hydrogenation
Source: Materials (Basel). 2024 Jun 21;17(13):3058. doi: 10.3390/ma17133058 (PMC11242047; doi:10.3390/ma17133058)
Supplement: Supplementary file 1 [file materials-17-03058-s001.zip › materials-3032985-supplementary.pdf]

Supporting information

Waste plastic-supported Pd single-atom catalyst for hydrogenation

Ziyue Wang, [1] Ying Zhang, [1] Hao Zhang, [1] Qingdi Sun, [1] Xiaohui He [1,3]* and Hongbing Ji [1,2]*

1. Key Laboratory of Bioinorganic and Synthetic Chemistry of Ministry of Education, Fine Chemical Industry Research Institute, School of Chemistry, Institute of Green Chemistry and Molecular Engineering, Sun Yat-sen University, Guangzhou 510275, China.

2. State Key Laboratory Breeding Base of Green-Chemical Synthesis Technology, Institute of Green Petroleum Processing and Light Hydrocarbon Conversion, College of Chemical Engineering, Zhejiang University of Technology, Hangzhou, 310014, China.

3. Guangdong Technology Research Center for Synthesis and Separation of Thermosensitive Chemicals, Guangzhou 510275, China.

* *Correspondence: hexiaohui@mail.sysu.edu.cn; jihb@mail.sysu.edu.cn*

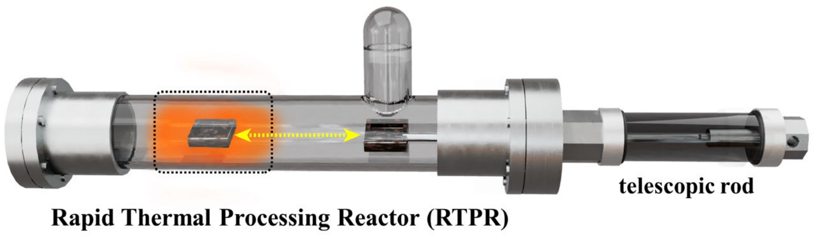

Figure S1. Schematic diagram of the Rapid Thermal Processing Reactor (RTPR).

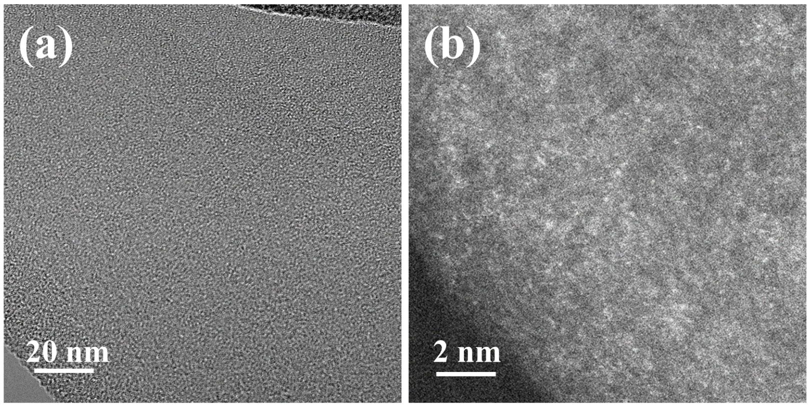

Figure S2. (a) TEM image and (b) AC HAADF-STEM image of Pd_1/PU after six cycles.
